# Optimum Expanded Fraction for an Industrial, Collins-Based Nitrogen Liquefaction Cycle

**DOI:** 10.3390/e22090959

**Published:** 2020-08-30

**Authors:** Carlos Arnaiz-del-Pozo, Ignacio López-Paniagua, Alberto López-Grande, Celina González-Fernández

**Affiliations:** 1 ETSI Industriales, Universidad Politécnica de Madrid (UPM), José Gutiérrez Abascal 2, 28006 Madrid, Spain; celina.gonzalez@upm.es; 2 Consejo Superior de Investigaciones Científicas (CSIC), Instituto de Cerámica y Vidrio. Kelsen 5, Campus de Cantoblanco, 28049 Madrid, Spain; alberto.lopez@icv.csic.es

**Keywords:** large-scale Collins cycle, thermodynamic analysis, exergy analysis, optimisation, nitrogen liquefaction, exergy efficiency, optimum expander flow, specific power consumption, liquid yield

## Abstract

Industrial nitrogen liquefaction cycles are based on the Collins topology but integrate variations. Several pressure levels with liquefaction to medium pressure and compressor–expander sets are common. The cycle must be designed aiming to minimise specific power consumption rather than to maximise liquid yield. For these reasons, conclusions of general studies cannot be extrapolated directly. This article calculates the optimal share of total compressed flow to be expanded in an industrial Collins-based cycle for nitrogen liquefaction. Simulations in Unisim Design R451 using Peng Robinson EOS for nitrogen resulted in 88% expanded flow, which is greater than the 75–80% for conventional Collins cycles with helium or other substances. Optimum specific compression work resulted 430.7 kWh/ton of liquid nitrogen. For some operating conditions, the relation between liquid yield and specific power consumption was counterintuitive: larger yield entailed larger consumption. Exergy analysis showed 40.3% exergy efficiency of the optimised process. The exergy destruction distribution and exergy flow across the cycle is provided. Approximately 40% of the 59.7% exergy destruction takes place in the cooling after compression. This exergy could be used for secondary applications such as industrial heating, energy storage or for lower temperature applications as heat conditioning.

## 1. Introduction

Liquefying gases is a very energy demanding process. The further away the temperature and pressure of a stream or system are from ambient temperature and pressure ($T_{0},P_{0}$), the higher its exergy. In a liquefaction process, a substance in gaseous state is taken from ambient conditions to a thermodynamically distant state in which it becomes liquid. Therefore, the exergy of the substance at the end of the process will be substantially high.

In this sense, the process of liquefaction is an injection of exergy. Starting from a gaseous state, the substance is given exergy by the liquefaction facility, a compressor-driven thermodynamic cycle designed to remove its sensible and latent heat. Very low temperatures must be reached in order to produce liquid, which in the case of nitrogen can be in the order of 77 K, approximately $-196{\;}^{\circ }$C.

Removing heat at temperatures so far from the ambient is necessarily exergy consuming. It can be observed in Equation (1) that the input of exergy, ΔB, required per unit of heat removed, *Q*, increases steeply the further temperature *T* is from the ambient, $T_{0}$:

$$\Delta B=Q\left(1-\frac{T_{0}}{T}\right)$$

A thermodynamic cycle for gas liquefaction can be designed around a basic principle based on two flows of gas. The flow of gas which is going to be partially liquefied, “forward flow”, must be at a high pressure. It is cooled down and then expanded at a Joule–Thomson valve coming out as a two-phase stream. The liquid fraction is the product and is extracted from the process. The remaining vapour, which is cold and at a low pressure after expansion, is used to cool down the forward flow. This flow constitutes the “return flow”.

The most direct realisation of this principle is the Linde–Hampson process, which is used for low-scale applications. Liquid air energy storage (LAES) systems consist of an air liquefaction unit for charging a liquid air reservoir and a power unit for discharging it. An analysis of a LAES system based on a modified Linde–Hampson cycle is presented in [1]. It includes a pre-cooling unit capable of extracting heat out of the process. The system is analysed operating with different degrees of pre-cooling: from the original configuration without pre-cooling to up to 200 K pre-cooling. An increasing trend in the liquid yield is clearly observed. This topology operates at high pressures reaching an optimum liquid yield of approximately 11% at 260 bar. It is concluded that the efficiency of the process can be improved with a Claude topology.

A Claude cycle [2] includes an expander connecting the forward and return flows of the Linde–Hampson cycle at an intermediate stage of the cooling. Part of the mass flow is kept on the forward flow and part is diverted to the expander. In the expander the pressure and temperature of the expanded mass flow will drop producing work, thus allowing to recover a fraction of the compression duty. The expander outlet is mixed with the returning mass flow of the vapour fraction after expansion, increasing the overall cooling capacity from this point on. With the addition of the expander the Claude cycle operates at significantly lower pressure and produces a higher liquid yield than the Linde–Hampson. A Claude LAES system is analysed in [3] operating at 40 bar reaching a liquid yield of 22%.

Minor modifications [4] soon become better options when specific power consumption requirement (per unit mass of liquefied gas) and specific investment costs are considered even at medium scale. The Kapitza topology is a modification of the Claude by which the expander is connected in parallel to the expansion valve [5], simplifying the design. In [6], several liquefaction topologies for medium scale cryogenic energy storage (CES) are compared technically and economically: Linde–Hampson, pre-cooled Linde–Hampson, Claude and pre-cooled Kapitza cycles. The pre-cooled Kapitza topology shows very high performance in terms of liquid yield and results the better option from an economic perspective, presenting the lower investment cost and being the most cost effective process. A sensitivity analysis shows a strong dependence of the power consumption per unit of liquid product (specific power consumption (SPC)) on the ratio of the total mass flow diverted to the expander in the Claude and Kapitza cycles. This last shows an optimum at approximately 38% of expanded mass flow.

Large-scale plants for gas liquefaction are based on Kapitza or Collins topologies as indicated in [7]. Instead of combining a pre-cooling unit with an expander, the Collins topology is a modification of the Claude cycle using two expanders [8].

**Figure 1:**
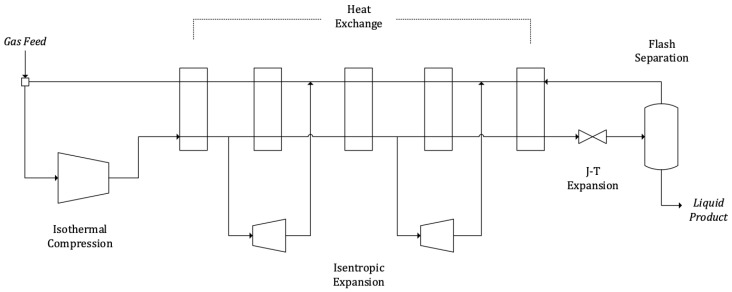
Diagram of the standard Collins cycle.

A diagram of the standard Collins cycle is shown in Figure 1. If no mass flow was diverted to the expanders the cycle would operate as a Linde–Hampson. If only one of the expanders was used it would operate as a Claude. Calculating the ratios of mass flow diverted to each of the expanders that minimise SPC is a core design problem.

The gas is first compressed isothermally in C (in a series of adiabatic compression–after-cooling stages), then pre-cooled in E1. After E1 the flow is split. A fraction is diverted to expander Ex1. The remaining flow is further cooled in E2 and E3. At this point it is split again and a fraction is sent to the downstream expander Ex2. The remaining forward flow continues cooling down in E4 and E5 and then flashed in the Joule–Thomson expansion valve. The flow exits the valve in two-phase into the vessel V where the liquid fraction is extracted. The vapour fraction, at low pressure and cold, is recycled into E5 after which it is mixed with the flow expanded in Ex2, heated in E4 and E3, then mixed again with Ex1 outlet and finally heated in E2 and E1. Finally the feed gas is introduced previous to recompression in C.

The temperature of the gas at the inlet of the J–T valve has been specifically indicated in Figure 1 by $T_{valv}$. The liquid yield will be greater the cooler $T_{valv}$, so the objective of an industrial cycle will be to achieve the lowest $T_{valv}$ possible with the lowest SPC.

The compromise between $T_{valv}$ and SPC is not straightforward. Two aspects must be taken into consideration: first, that the net power consumption of the cycle is the balance between the power consumption of the isothermal compression C, and the power produced at expanders Ex1 and Ex2. Second, that the efficiency of the heat exchange will determine $T_{valv}$. While the power consumption at C is defined by the pressure ratio between forward and return flows, the efficiency of the heat exchange is greatly dependent on the share of the total mass flow that is expanded at Ex1 and Ex2. This is because the changes in the mass flow ratio between the forward and return streams caused by diverting mass to the expanders determine their heat capacity ratios, and thus the temperature difference between the streams. In [9], the heat transfer process is studied for a helium liquefaction cycle. The study analyses the efficiency of the heat transfer process in depth, evaluating the effect of the expanded fractions in Ex1 and Ex2 (see Figure 1) on the efficiency of the heat exchange. It concludes that efficient heat exchange in E1 and E5 through optimum expanded mass flows must be ensured. The expanded mass flow ratio at Ex1 mainly affects E1, while the expanded mass flow ratio at Ex2 mainly affects E5. In [7], a discussion of the types of heat transfer in cryogenic cycles is provided. As commented in the text, the heat exchange at E5 may take place in the vicinity of the critical point when liquefying substances such as nitrogen. The specific heat will show large variations during the process, leading to an irregular temperature profile of the forward flow, so that the minimum temperature approach (MITA) in E5 will take place in between both ends.

Several studies analyse how the share of expanded flow affect the performance of a standard Collins cycle. In [10], the expanded mass flow ratios for minimum SPC are calculated for helium liquefaction. The optimum expanded mass flow ratios are 45% for Ex1 and 35% for Ex2, adding a total expanded mass flow of 80% of the total flow at compressor C. The total expanded mass flow ratio can be reduced if the heat exchange is increased, either by more effective heat exchangers or external pre-cooling, down to 75–79%. Similar results are obtained in a more recent study for an analogous Collins helium liquefaction cycle in [11]. Expanded flow ratios for maximum liquid yield are calculated, with similar assumptions to those of the previous study. The cycle is simulated with Aspen HYSYS across a range of expanded mass flow ratios. Although the main conclusion of the study is similar, that the optimum expanded mass flow is 80%, the resulting share between Ex1 and Ex2 is 50%, differing from the larger Ex1 ratio of the previous study. The article points out that results are similar for uneven distributions between the expanders up to 40–60% or 60–40%, and that distributions with higher Ex1 flow provide more greater liquid yield. The analysis of the heat process in this study, by the same author as [9], is coherent with the observations about the importance of E1 and E5 for the efficiency of the cycle concluded in [9].

As it has been mentioned, nitrogen liquefaction presents some particularities due to the large variations of the specific heat in the vicinity of the critical point. However, few specific assessments exist in the literature. In [12], a Kapitza cycle is simulated. The influence of diverting mass to the expander on the heat exchange process is identified, along the lines commented above and in [7]. A small-scale pre-cooled Kapitza cycle is analysed in [13]. Due to the scope of the study, the compressor discharge pressure is limited to 8 bar. Liquid yield results slightly over 6%. As the study mentions, this exceptionally low value would increase with higher compression ratios. The study provides the temperature profiles of the forward and return flows. It is interesting to observe that at 8 bar, well below the critical for nitrogen (33.9 bar), no irregularities due to large variations of specific heat can be observed. The large exergy losses in the aftercooler are identified as a major cause of exergy destruction, 26.03% of the total. The study [14] analyses an industrial pre-cooled Kapitza cycle for nitrogen liquefaction, operating at a compressor discharge pressure of 43.27 bar, sufficiently close to the critical point to observe the large variations in specific heat. The relation between heat transfer at E5 and the expanded mass flow ratio at Ex2 is assessed, analysing the variation of the temperature profiles for off-design operation.

In this article, an industrial Collins-based nitrogen liquefaction process is analysed in detail in order to find the optimum expanded flows that minimise SPC. The cycle does not correspond to the standard cycle of Figure 1. As any industrial cycle, it must be adapted to the requirements of the product and integrate modifications for large-scale liquefaction [15]. The high pressure level of the cycle is 36 bar, close to the critical 33.9 bar, so in contrast to the helium case, the large variations in specific heat mentioned earlier will affect the last stage of the heat exchange and appear clearly on the temperature profiles. Although the underlying Collins topology explains a similar behaviour of the studied cycle to the standard, the resulting SPC and optimum expanded mass flow show differences.

A detailed explanation of the cycle and its modifications over the standard Collins cycle will be developed in Section 2.1. The last two compression stages have been coupled to the two expansions in the cycle for better efficiency, as would happen in a real industrial cycle, using *companders* (explained in Section 2.1) instead of independent compressor stages and expanders [16]. The topology also integrates a subcooling unit at the end of the cycle, necessary to prepare the liquid product for transportation and distribution (to reduce boil-off). It consists in an additional heat exchanger and a second expansion valve, where the previously liquefied nitrogen is flashed again and subcooled. The low pressure gaseous fraction is recycled, which requires an additional compressor. The cycle has four pressure levels distributed in two forward and two return flows, instead of the two of the standard cycle. The pressure of the nitrogen gas feed to the process is also adapted to the typical industrial value of 4 bar, instead of the usual value employed in other studies of approximately 1 bar. Global assumptions and design parameters are described in Section 2.2.

Section 3 describes the series of simulations that have been carried out in Unisim Design R451. The expanded mass flows that minimise SPC have been calculated and a global optimum has been identified Section 3.1. This case has been analysed in depth and a brief sensitivity analysis to component efficiencies developed in Section 3.2.

An exergy characterisation of the process has been carried out in Section 3.3. The rational exergy efficiency of the process and the exergy destruction in each component have been calculated. A Sankey diagram illustrating the exergy flows in the process have been generated.

## 2. Methodology

**Figure 2:**
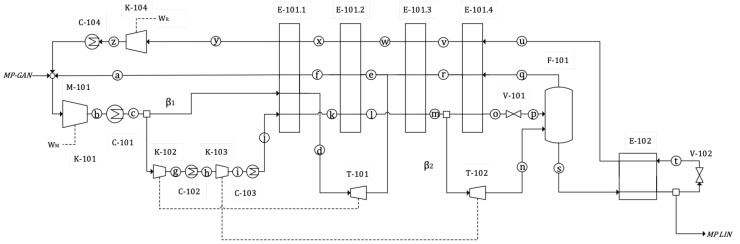
Diagram of the modified Collins cycle for nitrogen liquefaction. The cycle has four pressure levels and a bottom subcooler (E-102 + V-102). The flow ratios diverted to expander T-101, $\beta_{1}$, and to T-102, $\beta_{2}$, are indicated. The dashed lines indicate that T-101 and T-102 expanders are mechanically linked to K-102 and K-103 compressors.

Figure 2 shows the component diagram of the industrial N${}_{2}$ liquefaction cycle, which will be developed in detail in Section 2.1. The cycle will be simulated across a range of different mass flow ratios, $\beta_{1}$ and $\beta_{2}$, in order to find the lowest SPC configuration for a fixed set of process assumptions presented in Section 2.2. It is the same design problem as in [10,11] for helium but with a more complex topology in which the highest pressure level, HHP, is actually determined by $\beta_{1}$ and $\beta_{2}$, due to the use of *companders* (see Section 2.1).

**Figure 3:**
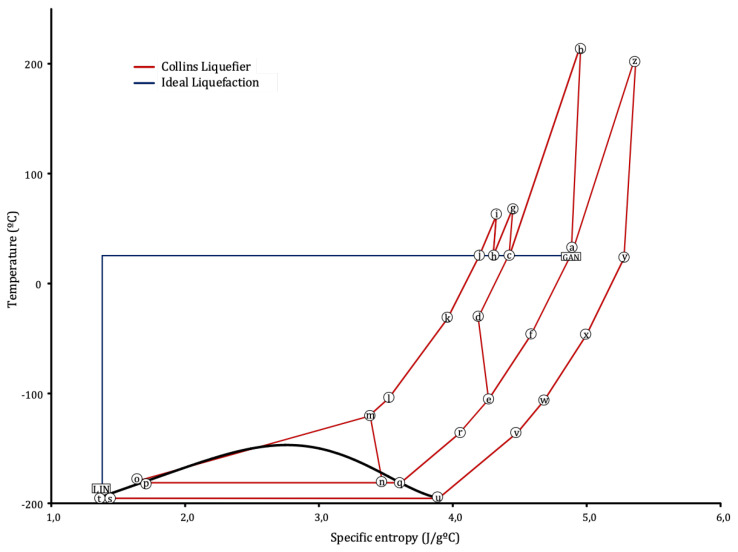
T−s chart for nitrogen. The ideal process leading to the theoretical minimum of specific power consumption (SPC) is represented in blue, the real process taking place un the cycle in red. The state labels of the component diagram of Figure 2 have been indicated.

The optimisation problem for this cycle and the role of the *companders* will be summarised in Section 2.3, in which the similarities with the underlying Collins cycle can be observed. The process is described in terms of exergy in Section 2.4, and a T−s diagram is shown in Figure 3.

### 2.1. Thermodynamic Cycle

The diagram of the cycle is shown in Figure 2. It can be observed that it is a modified Collins cycle with four pressure levels: LP, MP, HP and HHP. The main forward flow is ‘c’-‘p’, cooled by the MP and LP return flows (“q”–“a” and “t”–“z”, respectively). The flows diverted to expander T-101, $\beta_{1}$, and to T-102, $\beta_{2}$, increase the cooling power of the MP return flow. The main expansion valve is V-101, flashing two-state N${}_{2}$ into the F-101 vessel. A subcooler after F-101 is required to adapt the product for transportation. This is done with E-102 and a second expansion valve V-102.

A main N${}_{2}$ compressor K-101 with an aftercooler C-101 compresses the MP Nitrogen to HP level. It is then split into two fractions: The first one is cooled at the first cold box section, E-101.1 and expanded in T-101 to MP level into the MP return flow.

The second one is further compressed to HHP and cooled in K-102, C-102, K-103 and C-103. These compressors are powered by the expanders downstream, in *companders*, as will be explained later. The compressed gas leaving C-103 at state “j” is then cooled at E-101.1-3 and then split again, diverting ratio $\beta_{2}$ to expander T-102, where it expands to MP level into the F-101 vessel. The remaining HHP flow is further cooled at E-101.4 and finally flashed at the Joule-Thomson valve V-101 into the F-101 vessel.

The vessel F-101 is at MP level. There the liquid and vapour phases are separated. Vapour is returned to the start through the E-101 series of exchangers. The liquid fraction is subcooled at E-102, then split into two, one part is the actual dispatched product. The other stream is flashed to LP level at V-102, producing the temperature drop required for the subcooling, so after flashing it is returned to E-102 and from there, through the series of E-101 exchangers to the start. Logically, before it is integrated with the feed and MP return flows, it has to be compressed back to MP at K-104 and cooled at C-104.

In a *compander* set, compressor and expander blades are fixed to the same shaft forcing them to rotate together (there can exist two shafts coupled with a gearbox to adapt rotation speeds). The expanding flow will generate power through the expander section which in turn, via the shaft, will drive the associated compression stage. Both streams are independent so they can be connected to different processes altogether, or to different points within the same process. In this cycle, in order to use the power produced by the expanded mass flow compressor K-102 and expander T-101 are integrated into a *compander*. The same happens with K-103 and T-102, integrated into a second *compander*. In this way, the compression duty is provided by the power produced at a different point of the cycle directly, instead of passing through an intermediate conversion to electricity, making the process more efficient and avoiding electric motors and generators, thus reducing cost. An example of a nitrogen cryogenic cycle using companders can be found in [17].

From the point of view of analysing the cycle, however, *companders* introduce an additional restriction that does not exist in the original Collins cycle: the power of the downstream expansions equal the power of the corresponding compression stages upstream. Therefore, increasing the maximum pressure of the cycle cannot be done independently, as it will necessarily be associated with greater amount of gas expansion downstream. This will alter mass flows and operating temperatures affecting the heat transfer between forward and return flows, the key factor for cycle performance.

### 2.2. Assumptions and Cycle Parameters

The following assumptions have been made.

- The system is in steady state.
- Component efficiencies do not vary with pressure, temperature and mass flow rate.
- The cold box is adiabatic.
- Pressure drops in pipelines are negligible.

These assumptions are used in order to isolate the behaviour of the cycle from the particularities of any specific equipment, and they are common in directly related studies [10,11]. Constant component efficiencies allow designing the optimum cycle configuration, which will be the target of this work (analysing a given facility across its operating range would require variable component efficiency). Table 1 details the main component specifications. Polytropic efficiency was used because it is more representative of the actual technology level and its value does not depend on the pressure ratio. The efficiency of compander compressor stages is lower than in the main compressor, because the coupling with the expander will limit performance. Performance of heat exchangers is defined by manufacturer values of Minimum Temperature Approach (MITA), within the 0 to 5.36 ${}^{\circ }$C range specified in [18].

The feed is assumed to be 100% N${}_{2}$ at medium pressure, MP = 4 bar. Pressure levels correspond to LP = 1 bar, MP = 4 bar, HP = 18 bar and HHP resultant.

The simulations have been carried out with Honeywell Unisim R451. Nitrogen properties have been calculated using a modified Peng–Robinson equation of state. The original Peng–Robinson equation [19] showed a remarkable accuracy for nitrogen. A number of improvements have been developed since [20]. It is of widespread use for pure nitrogen [13,21] and nitrogen mixture [22,23,24,25] cryogenic processes. Unisim Design R451 uses a modified Peng-Robinson EOS specifically recommended for all components of air in the range of pressure and temperature conditions of this article [26].

### 2.3. Optimisation Problem

The specific power consumption of the process is given by the duty of the main compressor, K-101, and the LP return compressor, K-104. As it will be explained in Section 2.1, the other two compressors K-102 and K-103 do not influence SPC directly:

(1) $$\mathrm{SPC}=\frac{{\dot{W}}_{M}+{\dot{W}}_{R}}{{\dot{m}}_{MPLIN}}$$

where ${\dot{W}}_{M}$ and ${\dot{W}}_{R}$ indicate main and low pressure cycle compressor power consumption, K-101 and K-104, respectively, as will be developed in Section 2.1, and ${\dot{m}}_{MPLIN}$ indicates total liquefied mass flow.

As it has been introduced in Section 1, there are two opposing effects in the Collins cycle (it would equally apply to Kapitza and other cycles). They also exist in the industrial cycle analysed here, although some details must be taken into account.

The temperature at the inlet of the expansion valve V-101 (state “o” in Figure 2) will be indicated by $T_{valv}$ for clarity. It has to be as low as possible in order to maximise the liquid fraction, $Y_{L}$, after flashing (state “p”).

This temperature is achieved by cooling the gas from state “c” (after the main aftercooler, C-101) in the series of heat exchangers, in which the cold, return flow extracts heat from the main, forward flow from “c” to “o”. The return flow is formed by two major streams. At medium pressure (MP), the non-liquefied fraction (which should be as small as possible) into which the outlet from the two expanders are injected. At low pressure (LP), the outlet from the subcooler (E-102 + V-102).

The greater the return mass flow, the greater the cooling prior to the valve (state ‘o’ in Figure 2), which will lower $T_{valv}$. The return flow can be increased by diverting as much nitrogen as possible to the expanders. Due to the *companders* linking T-101 and T-102 with K-102 and K-103 (see Section 2.1), changes in expansion power will also lead to a higher HHP pressure. This effect will tend to move state “o” further left toward the liquid region in Figure 3, thus increasing the liquid yield, $Y_{L}$.

The optimisation problem consists in calculating the values of $\beta_{1}$ and $\beta_{2}$ which minimise SPC. In appearance, this would mean that as much mass flow as possible should be diverted to the expanders, which equals large values of $\beta_{1}$ and $\beta_{2}$. However, this has an opposite effect that will become significant over a certain limit. The greater the expanded fraction is, the less nitrogen mass flow at point ‘o’ left to flash in V-101. When it is flashed, the liquid fraction, $Y_{L}$, will be high but the actual mass flow small. So there must exist a certain ratio of the mass flows ($\beta_{1}$, $\beta_{2}$) that, when sent to the expanders, will generate enough cooling while maintaining a sufficient mass flow through the expansion valve and minimises SPC.

### 2.4. Exergy Fundamentals

The general exergy balance for a given open volume *j* at rest, with streams *i* entering or leaving through the boundary, during a time differential dt can be formulated [27]:

(2) $$dB_{j}=dQ_{j}\left(1-\frac{T_{0}}{T}\right)-\left(dW_{j}-P_{0}dV_{j}\right)-dI_{j}+\sum_{i}e_{i,j}dm_{i,j}$$

where $dB_{j}$ is the change in total exergy (exergy+mechanical exergy) of the control volume, $dQ_{j}\left(1-\frac{T_{0}}{T}\right)$ the exergy content of the heat received during the process, being *T* the temperature of the control volume and $T_{0}$ that of the environment, $\left(dW_{j}-P_{0}dV_{j}\right)$ the exergy content of the work performed by the control volume and $dI_{j}$ the exergy destruction in the control volume. $\sum_{i}e_{i,j}dm_{i,j}$ is the balance of exergy entering and exiting the control volume through all inlets and outlet flows, where $e_{i,j}$ is the exergy of flow *i*:

(3) $$e_{i,j}=h_{i}-h_{0}-T_{0}\left(s_{i}-s_{0}\right)$$

where $h_{i},s_{i}$ are specific enthalpy and entropy at flow *i*, respectively.

The liquefaction cycle analysed here can be taken as a control volume with one nitrogen input, MP GAN, and one output, MP LIN, referring to Figure 2. The minimum exergy required for the process would be the difference in flow exergy between these two states: 173.4 kWh/ton. This is achievable by the blue path [7] in the T−s chart for nitrogen shown in Figure 3, which consists of an isothermal compression and an isentropic expansion, both reversible thus having zero exergy destruction, but technologically impossible. The real process has also been represented in the figure. The exergy analysis developed in Section 3.3 will compare both processes.

In order to obtain the exergy breakdown of the process, Equation (2) has to be applied to all components, with the assumptions of Section 2.2. The resulting expressions for exergy destruction at each component are summarised in Table 2.

## 3. Results

In this section, the results of the simulations are shown. Section 3.1 presents the evolution of SPC and design variables with $\beta_{1}$ and $\beta_{2}$. Section 3.2 carries out a brief sensitivity study of SPC to different component efficiencies. Finally, Section 3.3 discusses the results in terms of exergy, and presents a Sankey diagram of the optimum case.

### 3.1. Parametric Study

**Figure 4:**
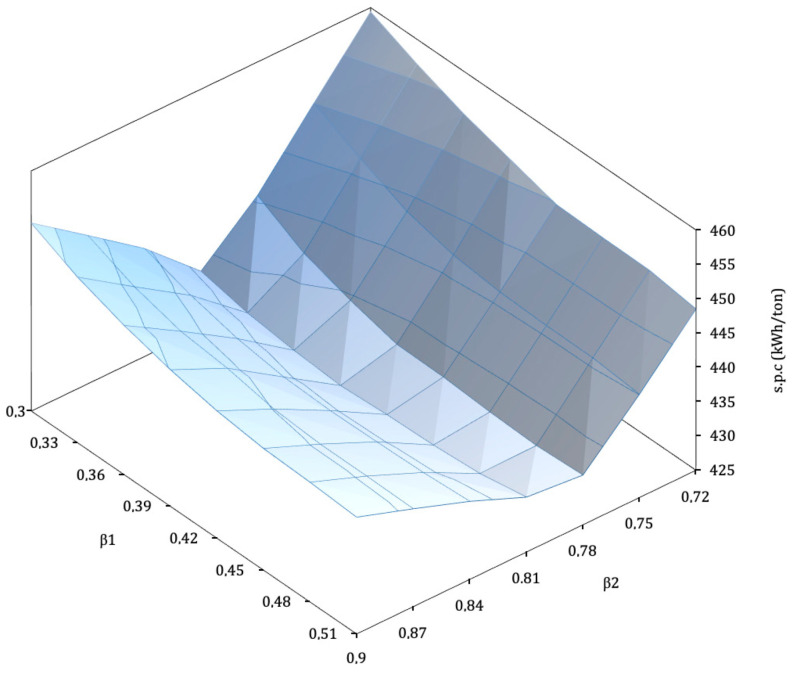
Specific power consumption (SPC) as a function of $\beta_{1}$ and $\beta_{2}$. The greater dependence of SPC with $\beta_{2}$ can be clearly observed.

The resulting optimum $\beta_{1}$ and $\beta_{2}$ add up to 88% of the total mass flow in K-101. The results for SPC (kWh/ton of LIN) across the range of $\beta_{1}$ and $\beta_{2}$ studied are given in Figure 4.

Different effects can be noted: for a given $\beta_{1}$, minimum SPC takes place at $\beta_{2}=0.81$. Power consumption rises steeply for $\beta_{2}$ away from the optimal. For lower values the temperature at the inlet of V-101 increases, as the smaller flow from the expander reduces the refrigerating power of the MP return flow, while at the same time more vapour is produced at the valve. The resulting MP return mass flow increases, therefore resulting in a higher SPC.

For higher $\beta_{2}$ values the flow through T-102 is high. This increases the refrigerating power of the MP return, but at the cost of flashing a smaller flow at V-102, thus leading to a low liquid mass flow produced. A small quantity of liquid abandoning the cycle means that the recycled mass flow is high, so recompression duty is high too.

**Figure 5:**
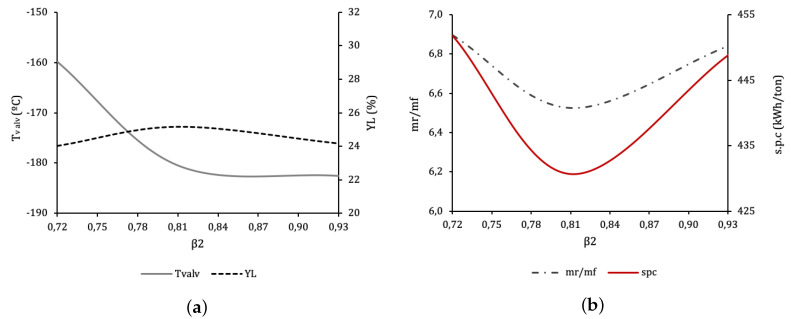
Different operation and performance parameters of the plant for the optimal $\beta_{1}=0.39$. (**a**) Temperature at V-101 expansion valve inlet, $T_{valv}$, and molar liquid yield at the F-101 expansion vessel, $Y_{L}$, as a function of $\beta_{2}$. (**b**) SPC and total recycle to feed mass flow ratio, $\frac{m_{r}}{m_{f}}$, as a function of $\beta_{2}$.

These effects can be observed in Figure 5, which shows the evolution of these variables when $\beta_{2}$ is changed from 0.72 to 0.93 for the optimal $\beta_{1}$. $T_{valv}$ is the temperature at the inlet of V-101 (state “o” in Figure 2). $Y_{L}$ is molar liquid yield in F-101. $\frac{m_{r}}{m_{f}}$ is the total recycle to feed streams ratio at M-101. Below the optimum, increasing $\beta_{2}$ increases the refrigeration power of the return flow at E-101.4, decreasing $T_{valv}$. It also increases compression power at K-103, which can be observed that tends to displace state “o” in Figure 3 further into the liquid region. As a result of both effects, state “p” will move left towards saturated liquid, increasing $Y_{L}$ and decreasing SPC. For $\beta_{2}$ above the optimum, the heat exchange process at E-101.4 restricts the variation of $T_{valv}$, so a greater $\beta_{2}$ will only tend to increase the pressure at “o”. This positive effect however, is lower than that of the mass flow at state “n”, the outlet of expander T-102. This is rich in vapour, so a greater mass flow will contribute negatively to $Y_{L}$ and increase the vapour mass flow which will have to be returned and compressed, therefore increasing SPC.

**Figure 6:**
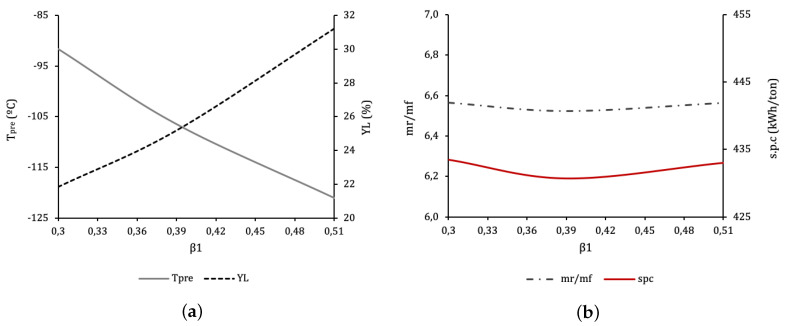
Several operating and performance parameters as a function of $\beta_{1}$, for optimum $\beta_{2}=0.81$. (**a**) Precooling temperature, $T_{pre}$ at state “l”, after the second heat exchanger section E-101.2 and liquid yield $Y_{L}$. (**b**) Ratio of recycle to feed mass flow $\frac{m_{r}}{m_{f}}$, and SPC.

Figure 6 illustrates the evolution of cycle parameters for a range of $\beta_{1}$ values, for the optimum $\beta_{2}$. The optimal value is $\beta_{1}=0.39$, resulting in ${\mathrm{SPC}}_{min}=430.7$ kWh/ton of LIN. Lower $\beta_{1}$ leads to less cooling at the beginning of the process and thus to a higher pre-cooling temperature after E-101.2 (state “l” in Figure 2, $T_{pre}$). In order to maintain performance the cooling provided by T-102 should compensate, but the strong effect of moving $\beta_{2}$ out of the optimal leaves no margin, and so performance necessarily drops: the overall recycle stream increases at M-101.

An interesting behaviour appears when $\beta_{1}$ is above optimal. Logically pre-cooling temperature $T_{pre}$ drops, increasing refrigeration and the liquid fraction with it. This increase, however, will not compensate the increase of the overall recycle stream caused by the larger flow through T-101. Having the liquid yield at F-101 increase at the same time as the SPC is totally counterintuitive.

**Figure 7:**
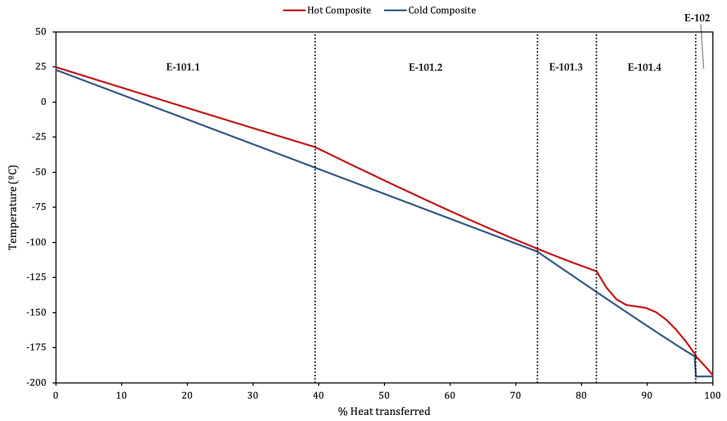
E-101 and E-102 heat-temperature profiles for optimal β values.

It is interesting to analyse the heat transfer between forward and return flows. The temperature profile at the series of heat exchangers E-101 and E-102 is shown in Figure 7 for the optimal $\beta_{1}$ and $\beta_{2}$. It is worth mentioning that almost 74% of the heat transfer occurs between E-101.1 and E-101.2.

Through E-101.1 and E-101.2, the straight profiles indicate a fairly constant heat capacity of hot and cold streams. The change in the slope of hot and cold composite curves is due to the different mass ratios of return/forward flows, given that the flow through T-101, which passes through E-101.1 HP hot side, returns by the MP cold side of E-101.2.

In E-101.3, the tendency is inverted again. The most relevant effect is the pinching of the temperature profiles that takes place at E-101.4. This phenomenon is also observed and explained in [7] for nitrogen at 40 bar, above the critical 33.9 bar. The pressure here is HHP = 36.0 bar, slightly closer to the critical. When the hot stream enters the exchanger at “m” (Figure 2), the heat capacity of the cold stream is much greater, so the temperature of the hot stream is more affected by the heat exchange taking place, decreasing steeply. This is the first converging stretch at E-101.4 in Figure 7. However, as the pressure of the hot stream is near the critical, minimal changes in temperature cause large changes of the specific heat. It rises steeply, achieving a maximum at −146 °C. The increase of specific heat equals an increase in the heat capacity of the stream, explaining the diverging stretch. As the temperature decreases further from that point the specific heat will decrease steeply too, and with it the heat capacity of the stream, until it will be lower than the cold stream capacity. Temperature profiles will converge again, as at the initial section.

It is worth mentioning that the relative disposition of the temperature profiles at E-101.4 has great relevance to the performance of the cycle. As it was mentioned in Section 1, this was also identified in [9] for helium and in [14] for nitrogen in a Kapitza cycle. It can be seen in Figure 7 how achieving the smallest temperature difference at the hot end of E-101.4 will minimise the temperature differences at E-101.3, E-101.2 and E-101.1, achieving a better match of the profiles. Therefore, the minimum approach in E-101.4 must be sought at the outlet, and at the point where the profiles change from converging to diverging (neighbourhood of maximum specific heat). This can be controlled by $\beta_{2}$, which determines the heat capacity of the cold stream, and hence the relative inclination of both profiles across E-101.4. This explains the significance of this parameter (see Figure 5).

Regarding the helium Collins cycle analysed in [9], it is interesting to remark that the helium gas stream does not undergo the large heat capacity variations shown in this cycle, because the considered pressures are always >12 bar, therefore being well over the critical of 2.27 bar. The temperature profile of the last heat exchange will therefore be similar to that of E-101.2 in Figure 7, allowing a better profile matching and a lesser influence of $\beta_{2}$ in the performance.

The last section in the figure represents E-102, where the level cold curve shows the effect of the vaporisation of the return flow taking place in the cold side for subcooling the MP LIN product.

### 3.2. Sensitivity Analysis

A sensitivity analysis of the SPC to efficiency of the main components of the process was carried out. The temperature approaches in E-101, polytropic efficiencies of main compressor K-101 and expansion devices T-101 and T-102 (denoted by $\eta_{p}$) were varied as described in Table 3. The sensitivity kept the previously obtained optimal $\beta_{1}$ and $\beta_{2}$ values constant, as no variation of the optimal $\beta_{2}$ was observed for the simulations with different efficiencies from the base case, while changes in SPC for B1 variations was minimal. These results showcase the relative independence of optimal expanded fraction with respect to component efficiencies in the design phase of the cycle.

**Figure 8:**
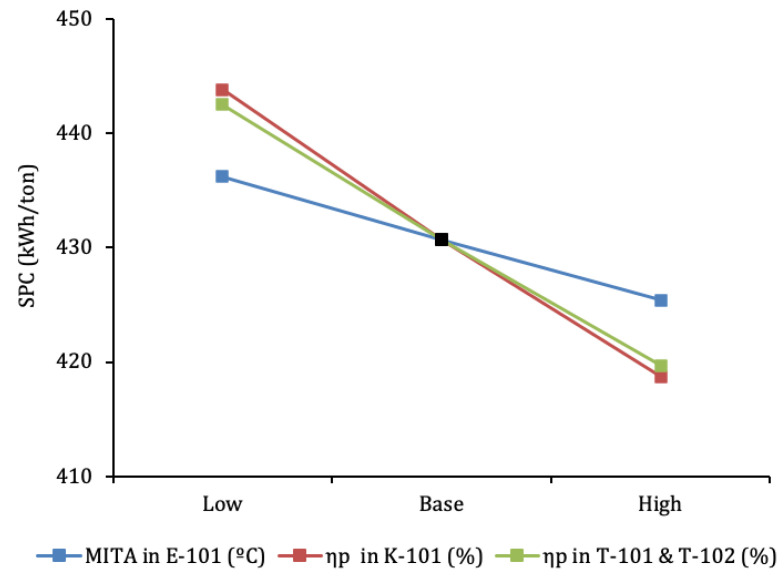
Sensitivity analysis: effect of component efficiencies on SPC.

The results of the sensitivity evaluation are shown in Figure 8. It can be seen that the influence of the efficiencies of the compressor and the expanders is similar. This seems a logical conclusion, given that SPC is the result of the balance between the power consumed by K-101 and the power generated by T-101 and T-102. On the other hand, the influence of the MITA in E-101 is lower than that of the compressor and expanders for the range of values covered. This points to the heat exchange process discussed in the previous section. The temperature profiles at E-101.4 do not allow a good match, which will inevitably have a large temperature drop at E-101.4 inlet (states “m” and “r”), whose effect will dominate over slight improvements of MITA.

### 3.3. Exergy Analysis

As it was mentioned in Section 2.4, the minimum specific exergy required for liquefying nitrogen for the given initial and final conditions would be 173.4 kWh/ton. This would result from the ideal, reversible process indicated by the blue path in the T−s diagram of Figure 3.

The real process, shown in red in the figure, differs greatly from the ideal one. It can be observed in Figure 3 how the temperature at the compression stages rise well above the isothermal path, reaching states “z”, “b”, “g” and “i”. This will increase the average heat rejection temperature of the cycle, thus increasing the total temperature drop and decreasing efficiency.

**Figure 9:**
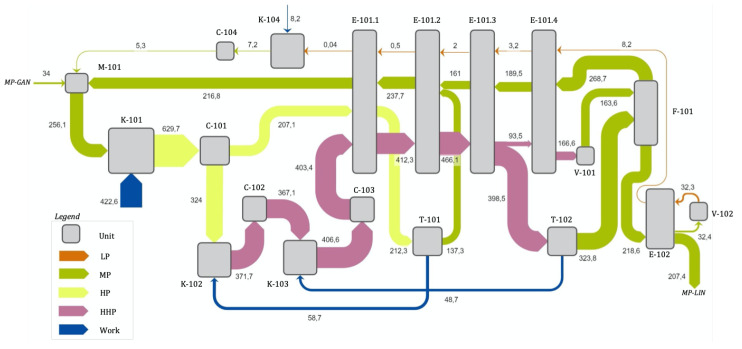
Exergy flow (Sankey) diagram of the process. Values are in kWh/ton of liquid N${}_{2}$. Exergy inputs and outputs at each component have been indicated. Blue arrows indicate work flows, the other arrows indicate exergy of the nitrogen streams, distinguishing pressure levels with colours. Especially remarkable is the exergy loss at aftercooler C-101 after the main compression, where heat is dissipated into the environment (dead state).

In addition, exergy destruction will take place in each component, ${\dot{I}}_{j}$, due to irreversibilities. An exergy flow diagram (or Sankey diagram) of the process is shown in Figure 9, where exergy inputs and outputs have been indicated. Exergy destruction is due mostly to fluid friction in turbomachinery and temperature drop between currents in heat exchangers (a full assessment of exergy losses in heat exchangers can be consulted in [28]). There will also take place exergy destruction due to heat rejection in the aftercoolers, especially aftercooler C-101 after the main compressor, where the total mass flow of hot nitrogen will release heat to a significantly cooler environment. As a result, the actual exergy required by the cycle is instead 430.7 kWh/ton at the optimum.

**Figure 10:**
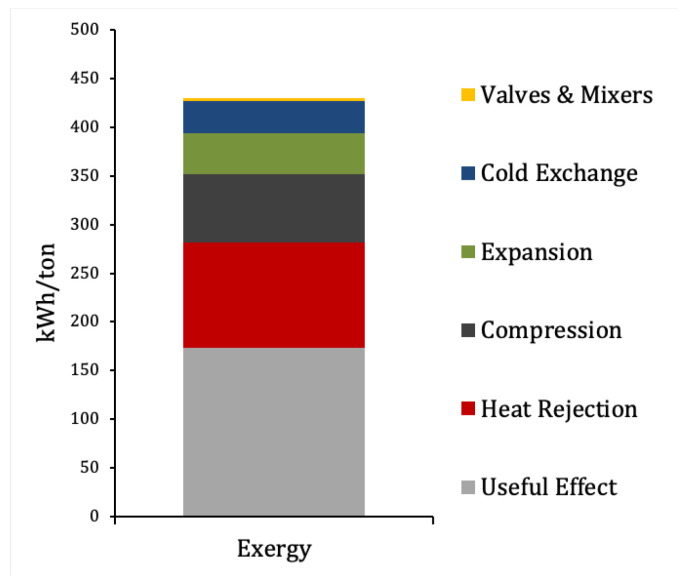
Total exergy breakdown per type of process of the optimised $N_{2}$ liquefier simulation. The contribution of valve expansion and mixing are low because the temperatures of the mixing streams are similar, and the mass flow at the valve is relatively small. On the opposite side, the contribution of compression and expansion processes—involving much greater mass flows—is large. Given the temperature range of the cold exchange, it is remarkable the moderate contribution to the total exergy destruction, due to the good matching of the temperature profiles throughout the series of heat exchangers. The contribution of aftercoolers is the largest.

**Figure 11:**
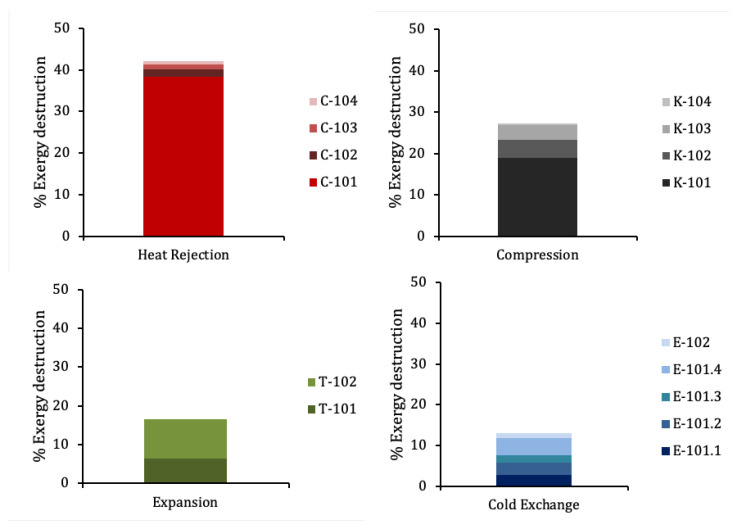
Exergy destruction distribution by component of the optimised $N_{2}$ liquefier.

The ratio between the minimum and the optimum SPC gives the exergy efficiency of the process, 40.3%. The remaining 59.7% corresponds to exergy destruction taking place in the different units. Figure 10 offers a detailed exergy breakdown of in absolute value (kWh/ton) per equipment type. Figure 11 shows the exergy destruction distribution per component in %.

It stands out in Figure 10 that 25.2% of the total exergy is destroyed at the aftercoolers, the single highest contribution to exergy destruction. This exergy is released to the environment (25 ${}^{\circ }$C) from the hot nitrogen after compression (up to 214.5 ${}^{\circ }$C). The large temperature drop and the mass flow at C-101 explain its magnitude, approximately 40% of the total exergy destruction. If this exergy were used, the exergy efficiency could increase up to 65.8%. Although the exergy of the rejected heat could not be recovered completely, a significant increase in overall efficiency could still be achieved. This points to the potential of using this exergy for other applications. A perspective of the potential of rejected heat from industry for district heating in the UK can be found in [29], where interesting considerations are developed on the distance from the heat source to the district network among other topics. A possible way to reduce the exergy loss at the compression is to perform the process in several stages with intercooling, to prevent temperature to rise excessively. However, this would increase investment costs depending on the number of compression and intercooling stages. A careful assessment should be carried out taking into account the electricity price curve, the hours of operation of the plant and the power consumption of the plant to ensure economic viability.

The rest of components of exergy destruction can be considered unavoidable or near the minimum physically attainable. The small share of destruction in valves may stand out given the intrinsic irreversibility of these processes, but this is explained by an equally small fraction of the mass flow being actually flashed.

Regarding helium liquefaction with a standard Collins cycle, the difference in cycle efficiency stands out, being in the order of 7% for helium [9] and 40% for this cycle. Several factors have to be taken into account. The lower density of helium will require greater compression power. The pressure ratio in [9] is 14, while the main compressor in the cycle analysed here is 4.1, thanks to the last stages being connected to the expanders downstream. Both factors will make the exergy loss greater for the helium process, because more heat will have to be dissipated to the environment. A value of 54% is indicated in [9] against 25% in this cycle.

An interesting remark can be made regarding the cold exchange in both cycles. Nitrogen reaches a minimum temperature of approximately 70 K in the process, while helium descends up to just over 4 K. As it was mentioned, the exergy destruction caused by any inefficiency is greater the lower the temperature, which would greatly penalise helium. According to the authors of [9], however, exergy destruction in the heat exchange for helium is approximately 10% and here it amounts to approximately 8%, which does not appear to be proportional to the difference in temperature. This can be explained by the better match of the temperature profiles for the case of helium, especially at E-101.4, which was commented in Section 3.1.

A final observation is that exergy destruction at the expansion valve for helium which in [9] amounts to 4.4%, while in this cycle it is approximately 0.7%. Again, it has to be taken into account that expansion takes place at a much lower temperature in the helium case and that the actual mass flow being expanded at the valve will be lower in the nitrogen case because of the larger expanded flow in the turbines.

## 4. Conclusions

An industrial $N_{2}$ liquefier based on a Collins cycle was analysed for optimum operating conditions. Minimum specific power consumption was estimated at 430.7 kWh/ton, with exergy efficiency of 40.3%. There exists an overall similarity in the behaviour of standard Collins cycles for helium liquefaction and the cycle presented in this study, motivated by the similar underlying phenomena. The optimum total expanded flow for this cycle is 88%, while for helium it results 75–80% [10] and 80% [11].

- The sensitivity of SPC was clearly higher to deviations of $\beta_{2}$, the downstream expander flow ratio, than to the upstream $\beta_{1}$. This sensitivity occurs because $\beta_{2}$ determines de efficiency of the heat transfer taking place at lower temperatures.
- The behaviour for higher than optimum $\beta_{1}$ is counterintuitive: liquid yield increases, but SPC is higher too, because of the larger recycle flow induced.
- A sensitivity analysis of the SPC to the main equipment efficiencies revealed that the efficiency of the expanders and of the main compressor have a greater influence than the MITA in the cold box within the range covered by the analysis.
- Integrating large liquefaction plants into district heating networks could allow using the exergy currently dumped to the environment in the form of heat rejection. Many industrial applications require heat in the 90 to 150 ${}^{\circ }$C range, which a Collins cycle could comply with.

This study covers optimum design parameters. Two interesting analyses have been identified for future work. First, to study an existing plant when operating in part load, identifying the optimal $\beta_{1}$ and $\beta_{2}$. Second, to study the behaviour of the optimal plant when forced to operate with non-optimum $\beta_{1}$ and $\beta_{2}$. Preliminary studies that have been carried out show that the effect of $\beta_{1}$ and especially $\beta_{2}$ are critical. The general trends shown in Figure 5 and Figure 6 hold around the design conditions for a wide range of operating conditions, but extreme values of $\beta_{1}$ and $\beta_{2}$ will bring the exchangers near their performance limits, altering the operation completely and thus defining the limits of operation of the plant globally. This is in line with the authors’ experience in the industry.

## Figures and Tables

**Table 1:** Technical parameters of the cycle elements.

Element	Parameter	Value	Unit
K-101, K-104	Polytropic efficiency	85	%
K-102, K-103	Polytropic efficiency	80	%
T-101, T-102	Polytropic efficiency	84	%
E-101	MITA	2	${}^{\circ }$C
E-102	MITA	1	${}^{\circ }$C
E-101	Pressure drop/side	20	kPa
E-102	Pressure drop/side	1	kPa
Aftercooler	Pressure drop	20	kPa

**Table 2:** Component nomenclature, equipment type and expressions for exergy destruction for each component type, obtained from the general balance of Equation (2).

Equipment Label	Equipment Type	Exergy Destruction, $\dot{I}$
K-101, K-102, K-103, K-104	Compressor	$\dot{m}\left(e_{in}-e_{out}\right)-{\dot{W}}_{c}$
T-101, T-102	Expander	$\dot{m}\left(e_{in}-e_{out}\right)-{\dot{W}}_{e}$
C-101, C-102, C-103, C-104	Cooler	$\dot{m}\left(e_{in}-e_{out}\right)$
E-101.1, E-101.2, E-101.3, E-101.4, E-102	Multi Stream Heat Exchanger	$\sum_{in}{\dot{m}}_{i}e_{i}-\sum_{out}{\dot{m}}_{i}e_{i}$
M-101	Mixer	$\sum_{in}{\dot{m}}_{i}e_{i}-m_{out}e_{out}$
F-101	Flash Vessel	$\sum_{in}{\dot{m}}_{i}e_{i}-\sum_{out}{\dot{m}}_{i}e_{i}$
V-101, V-102	Joule-Thompson Valve	$\dot{m}\left(e_{in}-e_{out}\right)$

**Table 3:** Component efficiency values for the sensitivity analysis.

Component/Efficiency	Low	Base	High
MITA in E-101 (${}^{\circ }$C)	3	2	1
$\eta_{p}$ in K-101 (%)	83	85	87
$\eta_{p}$ in T-101 & T-102 (%)	82	84	86

## Abbreviations

The following abbreviations are used in this manuscript:

ASU	Air Separation Unit
CES	Cryogenic Energy Storage
GAN	Gaseous Nitrogen
HP	High Pressure (bar)
HHP	Very High Pressure (bar)
LAES	Liquid Air Energy Storage
LIN	Liquid Nitrogen
LP	Low Pressure (bar)
LNG	Liquefied Natural Gas
MITA	MInimum Temperature Approach (${}^{\circ }$C)
MP	Medium Pressure (bar)
SPC	Specific Power Consumption (kWh/ton)
Symbols	
$T_{0}$	Ambient Temperature (${}^{\circ }$C)
β	Flow Fraction (–)
$e_{i}$	Exergy Flow (kJ/kg)
*W*	Work (kJ)
*Q*	Heat (kJ)
*I*	Exergy Destruction (kJ)
*B*	Exergy (kJ)
*V*	Volume (m${}^{3}$)
$h_{i}$	Specific Enthalpy (kJ/kg)
$s_{i}$	Specific entropy (kJ/kgK)
$T_{valv}$	Temperature before V-101 (${}^{\circ }$C)
$T_{pre}$	Temperature after T-101 (${}^{\circ }$C)
